# The mental health, quality of life and life satisfaction of internally displaced persons living in Nakuru County, Kenya

**DOI:** 10.1186/s12889-015-2085-7

**Published:** 2015-08-06

**Authors:** Elijah Mironga Getanda, Chris Papadopoulos, Hala Evans

**Affiliations:** University of Leicester, School of Psychology, The Greenwood Institute of Child Health, Leicester, LE3 0QU UK; University of Bedfordshire, Institute for Health Research, Putteridge Bury Campus, Bedfordshire, LU2 8LE UK

**Keywords:** Nakuru, Internally displaced persons, Mental health, Psychological distress, Quality of life, Life satisfaction

## Abstract

**Background:**

Internally displaced persons (IDPs) are among the most vulnerable people in the world today. Previous research highlights that conflict-induced forced displacement can cause problems with mental health and wellbeing. This study aimed to contribute to this body of knowledge by investigating the mental health, quality of life, and life satisfaction among IDPs living in Nakuru, Kenya.

**Methods:**

A questionnaire that included the General Health Questionnaire-12, Satisfaction with Life Scale, and a modified version of the WHO Quality of Life-BREF tool was used for data collection. The questionnaire also included an open-ended question inviting qualitative responses about their experience as an IDP. The questionnaire was distributed through a three-stage sampling approach across four refugee camps from four regions of the Nakuru County in Kenya.

**Results:**

One hundred IDPs participated in this study. All participants scored substantially higher than the applied GHQ-12 threshold for caseness (mean GHQ-12 score = 28.7, SD = 3.6). Quality of life and life satisfaction scores were also very poor (M = 10.24, SD = 1.9; M = 6.82, SD = 1.5 respectively). The qualitative results reflected these findings with statements reflecting suicidal thoughts, unhappiness with the government, lack of support, and fear for themselves and their children. Significantly higher GHQ-12 scores were found among older IDPs (rho = .202, sig = .046), widowers compared to married IDPs (mean difference = −2.41, SE = .885, sig = .027), while lower scores were found among IDPs who reported having friends as a source of support (U = 834, sig = .045), while quality of life scores were higher among IDPs who reported receiving governmental support (U = 248, sig = .018).

**Conclusion:**

The findings revealed poor levels of mental health, quality of life and life satisfaction. Older, widowed IDPs and those who did not perceive support from friends or the government were found to be at the highest risk of poor health and wellbeing.

## Background

Internally displaced persons (IDPs) are among the most vulnerable people in the world today [1]. The number of IDPs suffering globally stands over 26.4 million [2] with the majority living in low-income countries [3, 4]. At least two-thirds of the countries in Africa have experienced conflict leading to displacement of millions of people [5]. For instance, Kenya, a low-income, food-deficient country [6] is ranked 7th amongst countries with high numbers of IDPs in Africa due forced migration resulting from cultural inter-clan conflicts, social/communal tensions, politically influenced violence, and Government evictions [7, 8]. One such example of this was the 2007–08 Kenyan crisis which resulted in approximately 1,300 individuals killed and 600,000 Kenyans displaced [9]. The crisis was triggered after incumbent President Mwai Kibaki was declared the winner of the presidential election held in December 2007. Supporters of Kibaki's opponent, Raila Odinga, claimed that the election was fraudulent, sparking rioting between the rival party supporters. The conflict also led some groups to act on long-lasting grievances over the ownership of land which further contributed to large-scale displacement, particularly in the Rift Valley and western Kenya [10]. Overall, an estimated 350,000 of the IDPs sought refuge (three quarters of whom were women and children) in 118 camps spread across the country while another 314,000 either integrated within communities or moved to their ethnic homelands in the hope of security [11].

Epidemiological evidence that the burden on mental health is higher in conflict and post-conflict areas of the world compared to regions with no conflict is compelling [12]. This includes areas that have experienced targeted ethnic violence and conflict as a result of civil and political unrest [13–16]. Poor mental health has been argued to be particularly prevalent among IDPs who are exposed to trauma due to political conflict and oppression, and subsequent forced displacement into camps often unequipped to ensure safety and meet basic health and social care needs [17]; factors that further perpetuate risk of mental disorders [18, 19] For example, a recent study of mental disorders among West Papuan refugees exposed to political persecution and living in settlements under conditions of extreme poverty and deprivation identified a range of trauma event experiences and ongoing stressors, functional impairment ranging from mild to extreme, and over a quarter of the study sample (n = 230) meeting diagnostic criteria for one or more mental disorders. This included (in order of prevalence) separation anxiety disorder, persistent complex bereavement disorder, panic disorder, post-traumatic stress disorder, generalised anxiety disorder and more [20]. The aim of this study was to add to this evidence base by investigating the mental health, quality of life and life satisfaction of IDPs affected by the 2007–08 Kenyan crisis. To our knowledge, no previous study has investigated these outcomes within this particular population. A range of previously reported significant explanatory factors of poor mental health and wellbeing among IDPs were also investigated, including background factors such as age [21, 22], gender [22–24] and marital status [23, 25], employment [21, 25], duration of displacement [22, 23], and perceived level of support from individuals and organisations [26–28].

## Methods

### Design

This study employed a mixed-methods approach towards collecting and analysing quantitative and qualitative data.

### Instruments

The questionnaire was prepared and delivered in English, one of the two official languages in Kenya. If individuals agreed to participate they self-completed the questionnaire. However, if approached participants were unable to read or write, or did not understand the English language, the researcher (EM) administering the questionnaire was available to verbally translate the questionnaire into Swahili or one of the two dialects local to the county, Kikisii and Kikuyu, if required. The questionnaire’s first section included socio-demographic and background questions including age, gender, occupation, number of children, marital status, when they were displaced, whether they were displaced from within or outside of the Nakuru district and what their sources of support for their health are. The second section comprised of a modified, 6-item shortened version of the 26-item WHO Quality of Life-BREF (WHOQOL-BREF) tool [29] which has been previously applied successfully in the Kenyan context [30]. The tool asks participants to rate, using a 4 point Likert scale (very much, a moderate amount, a little, not at all), how much medical treatment they need to function in their daily life, whether they have enough energy for everyday life and how satisfied they are with their sleep (from the physical quality of life domain), how healthy they feel their physical environment is, how safe they feel their daily life is (both from the environment domain) and how well they are able to concentrate in the things that they do (psychological domain). The third section comprised of the Satisfaction with Life Scale (SWLS) [31] a 5-item scale designed to measure global cognitive judgments of one’s life satisfaction using a 7-point likert scale. While this measure has been widely validated it has not previously been adopted in Kenya. The fourth section comprised of the widely used and validated General Health Questionnaire-12 (GHQ-12) [32] which is a general (non-psychotic) psychiatric morbidity screening instrument that has previously been validated for the Kenyan population [33] . The Likert scale scoring procedure of 0-1-2-3 with summed scores of 12/13 applied as the threshold for identifying caseness. This conservative threshold was selected as it is recommended for PTSD contexts [19]. The final section of the questionnaire asked the participants to provide any comments about their experience as an internally displaced person. This open-ended question enabled us to collect some additional qualitative narrative data about their experiences, quality of life, life satisfaction and health. Prior to collecting any data, participants were provided with an information form and signed informed consent was sought in all cases.

### Setting

The study was carried out in December 2012 and took place in Nakuru County in Kenya which has an estimated 1,760,174 inhabitants, 50.2 % of which are male and 16.2 % are aged under 5 years [34]. Nakuru County is one of the forty seven counties in Kenya and is located in the southeastern part of the Rift Valley Province. According to the Kenyan Ministry of Health [34] there are 136 public, 118 private, 13 nongovernmental and 52 faith-based health facilities in Nakuru and that, in public services, there are only 8 doctors, 69 nurses and 10 clinical officers (per 100,000 people), while only 26.6 % of the population are covered by the National Health Insurance Fund. Approximately 13.3 % of Nakuru County population are estimated to be underweight while 25.9 % are estimated to be stunted [34]. It is also estimated that 5.6 % of all adults and 0.5 % of all children living in Nakuru are living with HIV while 10.3 % of all households are living with an orphan [35]. Investment in health in Kenya largely focuses on communicable diseases with mental health among the country’s lowest priorities, representing less than 1 % of the total public health budget [36]. There are approximately only 25 public sector psychiatrists and 500 psychiatric nurses in the country for a population of 38 million [37], the majority of whom are concentrated in urban areas [38]. Consequently, mental health remains a tabooed and widely misunderstood topic, particularly in rural areas [39].

In 2008, the Kenyan Government decided to erect ‘transit camps’ to settle internally displaced people affected by the 2007 disputed presidential elections that had sparked violence in most parts of the country [11]. Over 30,000 IDPs were forced to settle among 43 camps [11] erected across four regions of Nakuru County, the fourth largest county in Kenya [10]. However the camps are severely overcrowded, unsafe and consist of poor quality camps many of which are small, tattered and leaking [40]. Due to overcrowding many IDPs must sleep on the floor without mattresses and blankets [41]. The camps have limited access to food, sanitation, clean drinking water, and educational and health services [8, 11]. These poor conditions have exposed IDPs to health and social problems that, coupled with adverse weather conditions, have led to such respiratory complications as asthma and pneumonia among the adults and children, family breakdowns and an increase in risky sexual health behaviours [41]. Reports have also highlighted the challenge in accessing facilities for mental health support due to a lack of provision [41], a lack of awareness [42], and facilities located too far away [43].

### Sampling

A three stage sampling approach was employed. Firstly, four camps (Tuinuane, Nawam, Jikaze and Minto) which housed 547 tents in total (see Table 1) from the four regions of the county (Molo, Gilgil, Naivasha and Rongai respectively) were purposely sampled to ensure representation from each geographical region. The second stage involved randomly selecting a tent as a starting point and then applying a sampling interval rule of 3 tents. Systematic sampling was selected to help ensure that each camp would be evenly sampled. The interval of three tents was purposefully selected for pragmatic reasons. Before approaching each third tent, a coin toss was made in order to randomly select whether a male or a female participant would be sought. This third stage was built in to the sampling strategy to try to increase the chances of equal participation between males and females since the 2012 census figures report an even male–female ratio in this population [34]. Any internally displaced adult residing in one of the four study camps aged (18–65 years) were eligible to participate in the study.

**Table 1:** Number of tents per camp, number of tents approached and response rates

	Number	Percent
Nawam camp		
Total number of tents	205	-
Approached tents	68	100
Participated	36	53
Refused	9	13
Unavailable	23	34
Tuinuane camp		
Total number of tents	177	-
Approached tents	58	100
Participated	35	60
Refused	14	24
Unavailable	9	16
Jikaze camp		
Total number of tents	145	-
Approached tents	47	100
Participated	25	53
Refused	15	32
Unavailable	7	15
Minto camp		
Total number of tents	20	-
Approached tents	6	100
Participated	4	67
Refused	1	17
Unavailable	1	17

### Data analysis

Study quantitative data were analysed using IBM SPSS (v.19; IBM Corp., Armonk, NY, USA). Data cleaning and checking were first conducted. A descriptive analysis examining the sample characteristics and central tendencies of the three study outcome measures (GHQ-12, SWLS and modified WHOQOL-BREF) was conducted. As the data collected was not accurately representative of the target population, inferential statistics using non-parametric tests were then carried out. When Kruskal-Wallis H-tests were carried out, Tamhane’s T2 post-hoc tests were also used to identify significant pairwise comparisons. This post-hoc test was selected as it is appropriate for non-parametric data and when sample sizes are small [44]. Narrative data collected from the open-ended survey question were analysed using thematic analysis that involved categorising the content of the data into themes that emerged from the data collected.

### Ethics approval

Ethical approval for the study was received on November 15^th^ 2012 from the University of Bedfordshire’s Institute for Health Research Ethics Committee (IHREC). Ethics approval was also sought from the Kenyatta University Ethical Review Committee who advised us that IHREC approval was sufficient. In addition, area village leaders and administrative officials reviewed the study protocol and data collection instruments, interviewed the lead researcher, and provided approval and permission prior to the commencement of data collection. All potential participants were provided a participant information form and signed an informed consent document if they were happy to volunteer to participate. A mental health information sheet was provided to each participant after the collection of each completed questionnaire. If an individual refused to participate they were also offered this information sheet. This document consisted of the contact details of the mental health services most closely available to their camp.

## Results

One hundred internally displaced persons were successfully recruited, 62 of whom were female and 38 male. Thirty-six IDPs were recruited from Nawam camp, 35 from Tuinuane camp, 25 from Jikaze camp and 4 from Minto camp. The study participants were drawn from 100 tents that agreed to participate. Seventy-nine other tents were also approached. However, 39 of these households refused to participate in the study while nobody was available to participate in the 40 other households during our sampling procedure. The response rate (which includes refusers and non-contacts) was therefore 55.9 %. A full breakdown of the camp and IDP characteristics can be viewed in Tables 1 and 2 respectively.

**Table 2:** Full breakdown of participant background characteristics

	Number	Percent
Gender		
Male	38	38
Female	62	62
Marital status		
Single	26	26
Married	42	42
Widowed	31	31
Have children?		
Yes	57	27
No	43	43
Place of displacement		
Within the county	25	25
Outside the county	75	75
Duration of displacement		
5 years	68	68
7 years	20	20
10 years	12	12
Occupation		
Employed	71	71
Unemployed	29	29
Age	Range	Median
	21-62	43

Forty-five participants stated that they receive support for their health from non-governmental organisations, 35 participants reported this support came from friends, 28 participants reported support from family members and only 10 participants reported receiving support from the government.

All participants scored substantially higher than the applied 12/13 GHQ-12 caseness threshold. Mean WHOQOL_BREF and SWLS scores were 10.24 (SD = 1.9) and 6.82 (SD = 1.5) respectively. A full breakdown of the central tendency values of the three study outcome measures can be seen in Table 3.

**Table 3:** Central tendencies of study outcome measures

	Modified WHOQOL_BREF (lowest possible QoL score = 6; highest possible QoL score = 24)	SWLS (lowest possible satisfaction score = 5; highest possible satisfaction score = 35)	GHQ-12 (lowest possible score = 0; highest possible score = 36)
Mean	10.24	6.82	28.7
SD	1.9	1.5	3.6
Median	10	7	29
Range	7-17	5-12	18-35

WHOQOL_BREF = World Health Organization Quality of Life-BREF scale

SWLS = Satisfaction with Life Scale

GHQ-12 = General Health Questionnaire 12

As can be seen in Table 4, no background variables were found to significantly associate with life satisfaction scores. However, IDPs who reported receiving governmental support for their health scored significantly higher QoL scores than those who did not report receiving this support (U = 248, sig = .018). GHQ-12 scores were found to significantly correlate with age (rho = .202, sig = .046), and associate with marital status (x2 = 6.84, sig = .033). Post-hoc tests revealed that widowers scored significantly higher GHQ-12 scores than married IDPs (mean difference = .2.41, sig = .027, SE = .885) although not when compared against single participants (mean difference = .436, sig = .826, SE = .936). IDPs who did not report receiving support for their health from friends were found to have significantly higher GHQ-12 than those who did report such support (U = 834, sig = .045). No other significant relationships between background variables and outcome variables were identified.

**Table 4:** Factors associated with outcome measures

	WHOQOL_BREF	SWLS	GHQ-12
	MR	MR	MR
Gender			
Male	53	48	49
Female	48	51	49
U	1053	1073	1098
Marital status			
Single	46	52	52
Married	49	43	57
Widowed	52	55	38
x^2^	1.6	4.5	6.8*
Have children?			
Yes	50	52	47
No	52	48	53
U	1171	1130	1009
Place of displacement			
Within the county	47	56	43
Outside the county	52	49	52
U	861	799	735
Duration of residing in camp			
5 years	53	51	52
7 years	45	44	35
10 years	47	59	59
x^2^	1.5	2.3	6.2
Occupation			
Employed	49	50	48
Unemployed	54	52	53
U	921	982	870
Source of help – family?			
Yes	49	49	56
No	51	51	47
U	956	953	775
Source of help – friends?			
Yes	51	55	42
No	50	48	54
U	1108	965	834*
Source of help – government?			
Yes	71	46	43
No	48	51	50
U	248*	407	379
Source of help – NGOs?			
Yes	50	51	53
No	51	50	47
U	1206	1194	1045
	rho	rho	rho
Age	-.020	-.115	.202*
WHOQOL_BREF	-	.047	-.057
SWLS	.047	-	-.243*
GHQ-12	-.057	-.243*	-

* = P < 0.05; MR = Mean Rank; U = Mann–Whitney U-Test;

rho = Spearman’s rho test; x^2^ = Kruskal Wallis H Test

WHOQOL_BREF = World Health Organization Quality of Life-BREF scale

SWLS = Satisfaction with Life Scale

GHQ-12 = General Health Questionnaire 12

Thirty-one participants provided additional qualitative comments about their experience as an internally displaced person from which the following seven themes emerged. The theme ‘unhappy with life as IDP’ represents participants’ ongoing dissatisfaction and sadness with life as internally displaced person living in transit camps, and the belief that life will continue to be unhappy for as long as they remain in transit camps. Example quotes that reflect this are “*I am worried about my future and my life in this camp*”, “*I am not happy with life in this place*”, and “*It is only God who knows when I will ever have a better life*”.

The ‘suicidal thoughts’ theme captures IDPs’ suicidal ideation which was associated with regret to being born and their ongoing psychological suffering. Example quotes that reflect this are: “*For how long will I live this life?*”, “*I regret why I was born*”, and “*Was I born to suffer?*”

Another theme was ‘unhappy with the Government’ which highlights IDPs’ dissatisfaction with the lack of support they have received from their Government and the view that political leaders’ primary motivation was power and votes and not the wellbeing of IDPs. Quotes from this theme include: “*Why are our leaders so quiet but when election come they start seeking votes from us?*”, “*Why are politicians eating our money?*”, and “*Why can’t the government help us please*?”

Related to the latter theme is ‘needing urgent help’ which represents a generalised, broader call for urgent help including ‘well-wishers’ and the acceptance that they cannot cope by themselves without additional support. Example quotes that highlight this include: “*I need help from well-wishers please*” and “*I do not have resources to help myself*”.

The ‘lack of health support’ theme captures IDPs particular call for health-related support including the need for medicines, treatments and more accessible treatment facilities. Participant statements such as “*I always miss my medication due to lack of money and access to hospitals*”, “*I need treatment but I can’t afford it please*” and “*We need a clinic centre nearer here please*” manifest this.

IDPs also expressed ‘living in fear’ which including the anxiety of being attacked and also the desire to return home but the fear in doing so. Example quotes of this theme included: “*I don’t sleep at night for fear to be attacked*” and “*I want to go back my farm but I am scared*”.

Finally the theme ‘worried for their children’ represents IDPs acute anxiety and fear over their children’s needs, including their wellbeing and safety, apprehension over their possible trauma and, related, their fear for their future. This is highlighted by the following example quotes: “*I am worried of the future of my children*”, “*My children lack food, fees, and clothes, please help me*” and “*My children are traumatised for what happened in 2007*”.

## Discussion

The results of this study highlight how poor this particular population’s quality of life, life satisfaction, and psychological health is. For example, 100 % of the sample scored above the GHQ-12 cut-off for caseness. The findings from the qualitative analysis reflected the quantitative results, with identified themes highlighting suicidal ideation, unhappiness with life, living in fear and being fearful of their children’s wellbeing. Wide-scale distress and poor quality of life has been reported in previous research investigating the wellbeing of IDPs although not quite to the same extent. For example, Sheikh et al. [45] examined the psycho-trauma among IDPs affected by post-election violent conflict in Northern Nigeria. Their cross-sectional survey of IDPs living in a camp in Kaduna state reported that 42.2 % of their sample had a PTSD diagnosis (as measured by the Harvard trauma questionnaire) and that 16.3 % were depressed (as measured by the composite international diagnostic interview). Makhashvili et al. [22] investigated patterns of mental disorders among individuals affected by crises in Georgia including IDPs from the 2008 ethno-political Georgian-Ossetian conflict. They found that 28.3 % of their sample had a mental disorder with 22.9 %, 9.9 % and 9.2 % suffering from PTSD (as measured by the Trauma Screening Questionnaire), depression (as measured by the Patient Health Questionnaire 9) and anxiety (as measured by the Generalised Anxiety Disorder 7 instrument) respectively. Using a GHQ-28 cut-off score of 11, Hamid and Musa [23] reported that 70 % of sampled IDPs living across camps in Darfur suffered from general psychological distress. Roberts et al. [46] assessment of the quality of life SF-8 measurement instrument within IDP camps located in northern Uganda reported overall physical and mental component summary mean scores of 42.21 (SD = 11.93) and 39.27 (SD = 12.83) both of which are considerably poorer scores than the general United States population [47]. While there are clear contextual differences with each crisis being unique in nature, overall the evidence from this study and previous literature support the notion that experiencing conflict-driven internal displacement increases the risk of poor mental health and reduced quality of life.

Also notable was that no participant scored higher than 12 on the SWLS measure, and that the overall mean score was only 6.82. This score is substantially lower than general population samples; for example, Glaesmer et al. [48] reported a SWLS mean score of 25.12 among a representative German general population sample, while Vasquez et al. [49] reported a mean score of 24.16 among a representative Spanish general population. This study’s mean score is also considerably lower than any of the mean scores reported in Pavot and Diener’s [50] review of SWLS population scores, the lowest of which was reported to be 11.8 (SD = 5.6) from Frisch’s [51] hospital inpatient sample of veterans. To our knowledge, this is the first study that has reported life satisfaction scores among an internally displaced population and therefore no comparisons with other IDP samples are possible.

The study also revealed that only ten participants reported receiving any help and support from the governmental for their health. This is again reflected in the qualitative results where expressions of unhappiness with the government, lack of health support, and the need for help were key themes that emerged from the analysis. The ten participants who did receive governmental support scored significantly better than others on our quality of life measure, suggesting that this source of support is an important means towards improving wellbeing. Just over a third of the sample reported receiving help from friends and these individuals scored significantly higher in terms of mental health. This is indicates that perceived social support plays a powerful role in protecting against poor mental health, even among IDPs which such poor mental health and wellbeing outcomes. Previous research also emphasises this phenomenon’s power in determining health. Ozbay et al. [52] argue that “positive social support of high quality can enhance resilience to stress, help protect against developing trauma-related psychopathology, decrease the functional consequences of trauma-induced disorders, such as posttraumatic stress disorder (PTSD), and reduce medical morbidity and mortality” (p35). For example, Gorst-Unsworth and Goldenberg [53] revealed that, among a sample of 84 Iraqi male refugees, poor social support was a stronger predictor of depressive morbidity than trauma factors. Studies by Mels et al. [26] and Siriwardhana and Stewart [27] both found evidence that community resilience and social support are key mediators between the forced migration experience and the subsequent impact this upon mental health. Siriwardhana et al’s [28] systematic review of resilience and mental health outcomes among forced migration concluded that high quality social support consistently associates with increased resilience and better psychological wellbeing across all phases of conflict-induced forced migration. It could also be interpreted that this study’s finding of widowers having significantly poorer mental health compared to married IDPs partly substantiates this, given that the loss of spouse represents a real loss in psycho-social support. It also increases the risk of further trauma [54–56], depression [57, 58] and loneliness [59].

The analysis also identified a significant positive correlation between age and GHQ-12 summed scores, thus indicating that older IDPs are potentially less equipped at coping with the burdens of forced displacement. Lopes Cardozo et al’s [21] study similarly reported a significant relationship between age and poorer overall mental health as measured by the GHQ-28 (sig = .001). Araya et al. [60], who randomly sampled Ethiopian IDPs living in Addis Ababa following displacement that began in the early 1990s, found that mental distress (as measured by the SCL-90-R) significantly associated with older age (sig = .019). Hamid and Musa’s [23] finding of a significant positive correlation between age and the GHQ-28’s social dysfunction domain also provides partial support. Hamid and Musa theorise that this may be “because older IDPs might find it more difficult for them to regain their social status that they had enjoyed in their original societies” (p283). They also theorise that it is possible that they were experiencing greater hardship prior to being displaced due to limited mobility and activity.

A number of previous research studies have also identified gender, employment status and duration of displacement as significant factors in explaining differences in mental health and wellbeing outcomes among IDP populations. However this study did not evidence these relationships. Perhaps most surprising is that there was no identified difference between any of the study outcome measures and gender despite a large evidence-base consistently reporting significantly poorer outcomes for female IDPs [22–25, 61] possible explanation for this is that the study sample size was too small and unbalanced to detect any statistical differences among these variables. Additionally, differences in duration of displacement may not have been identified possibly because all of the IDPs sampled in this study had already been displaced for at least 5 years and had therefore been experienced similar hardships and been exposed to similar levels of exposure to traumatic events compared to those IDPs who had been displaced for 7 to 10 years. Had we been able to sample a wider range of displacement periods, differences may have been detected. For example, Makhashvili et al. [22] detected significantly higher levels of mental disorders among ethnic Georgians internally displaced in the 1990s compared to those displaced in 2008. Another reasonable interpretation is that we should not necessarily expect to identify consistent risk factors of poor health outcomes across the IDP literature given the heterogeneity of (a) the conflicts and crises that trigger internal displacement, (b) sampled populations and (c) study designs, particularly in terms of measurement tools [14] all of which warrant caution between making direct comparisons between studies. There are a number of limitations in this study. The study sample is unbalanced particularly in terms of gender, despite the coin-toss sampling strategy designed to increase likelihood of balanced male and female participation. The key reason for this was because of security curfews that prevented us from being able to also sample tents in the evening when more males would have more likely been available to participate. Due to lack of funds, resources and lack of recruitment time, only four camps were sampled. This means that the sample size is unpowered, relatively small, and, although the camps were purposively sampled across all parts of the county, they were not randomly sampled. All of these issues reduces population representativeness and limits generalisability. The WHOQOL_BREF was made shorter to increase response rates but in modifying it the tool results in unknown and questionable validity and reliability. While the SWLS has been previously widely implemented in other settings, unknown tool validity and reliability exists for the Kenyan population. Additionally, while all of the study participants were able to read and write in English, we acknowledge that it is possible that some of the IDPs that refused to participate may have done so because they were felt unable to sufficiently understand and complete the questionnaire. While the researcher was on-hand to verbally translate the questionnaire if required, it remains unknown as to whether the reason why no such request was made was because of this issue. Therefore we cannot rule out the English-language bias exists in the study sample.

## Conclusions

Overall this study indicates that being younger, married, perceiving to receive social and governmental health are IDPs who are most protected from poor mental health and wellbeing. However, it is important to note that this study also highlights that even these individuals still reported very poor outcomes, and that, overall, the study’s population present poor mental health and wellbeing, including suicidal ideation and fear. The key implication of this study’s findings is that the type of conflict-induced forced internal displacement observed in this study is harmful for overall health and wellbeing. Individuals in these circumstances require urgent help and support to improve their current and longer term health and safety, in particular IDPs who fear for the welfare of their families, do not perceive social support, and who experience poor mental health. This represents a formidable challenge for social, general health and mental health services in such contexts where such significant and complex health and social care needs exist. The challenge is compounded by problems of equity of access, resource capacity and competence, stigmatisation and lack of awareness in service function and availability.

## Abbreviations

IDPs: Internally displaced persons
GHQ: General Health Questionnaire
WHOQOL-BREF: World Health Organization Quality of Life-BREF tool
SWLS: Satisfaction with Life Scale
IBM SPSS: International Business Machines Statistical Package for the Social Sciences

## References

[1] Office of the United Nations High Commissioner for Refugees. Internally Displaced People: on the run in their own land. [http://www.unhcr.org/pages/49c3646c146.html]

[2] Office of the United Nations High Commissioner for Refugees. Global Overview 2011. People internally displaced by conflict and violence. [http://www.unhcr.org/50f95f7a9.html]

[3] Roberts B, Damundu EY, Lomoro O, Sondorp E (2010). The influence of demographic characteristics, living conditions, and trauma exposure on the overall health of a conflict-affected population in Southern Sudan. BMC Public Health.

[4] Thapa SB, Hauff E (2012). Perceived needs, self-reported health and disability among displaced persons during an armed conflict in Nepal. Soc Psychiatry Psychiatr Epidemiol.

[5] Burke MB, Miguel E, Satyanath S, Dykema JA, Lobell DB (2009). Warming increases the risk of civil war in Africa. Proc Natl Acad Sci U S A.

[6] World Health Organization. Communicable disease risk assessment and interventions. Post-election emergency: Kenya. February 2008. [http://www.who.int/neglected_diseases/diseasecontrol_emergencies/EPR_DCE_2008_1rr%20.pdf]

[7] The International Federation of Human Rights. Massive internal displacements due to politically instigated ethnic clashes. [https://www.fidh.org/International-Federation-for-Human-Rights/Africa/kenya/Massive-internal-displacements-due]

[8] Internal Displacement Monitoring Centre. Kenya - IDPs’ significant needs remain as intercommunal violence increases. http://reliefweb.int/sites/reliefweb.int/files/resources/kenya-overview-dec2012.pdf]

[9] Refugee Consortium of Kenya in Partnership with the Danish Refugee Council. Behind the Scenes. Lessons Learnt from Developing a National Policy Framework on Internal Displacement in Kenya. [http://drc.dk/fileadmin/uploads/pdf/IA_PDF/Great_Lakes_PDF/BehindTheScenes_KenyaIDPReport.pdf]

[10] Humanitarian Policy Group. HPG Policy Brief 31. Crisis in Kenya: land, displacement and the search for ‘durable solutions’. [http://www.odi.org/sites/odi.org.uk/files/odi-assets/publications-opinion-files/2263.pdf]

[11] United Nations Office for the Coordination of Humanitarian Affairs - Kenya. Frequently asked questions on IDPs. [http://reliefweb.int/sites/reliefweb.int/files/resources/D925666D168061D64925753300256FA5-Full_Report.pdf]

[12] de Jong JT, Komproe IH, Van Ommeren M (2003). Common mental disorders in postconflict settings. Lancet.

[13] Shannon PJ (2014). Refugees’ advice to physicians: how to ask about mental health. Fam Pract.

[14] Steel Z, Chey T, Silove D, Marnane C, Bryant RA, van Ommeren M (2009). Association of torture and other potentially traumatic events with mental health outcomes among populations exposed to mass conflict and displacement: a systematic review and meta-analysis. JAMA.

[15] Quiroga J, Jaranson JM (2005). Politically-motivated torture and its survivors: a desk study review of the literature. Torture.

[16] Fazel M, Wheeler J, Danesh J (2005). Prevalence of serious mental disorder in 7000 refugees resettled in western countries. Lancet.

[17] Porter M, Haslam N (2005). Pre-displacement and post-displacement factors associated with mental health of refugees and internally displaced persons: a meta-analysis. JAMA.

[18] Koehn PH (2006). Globalization, migration health, and educational preparation for transnational medical encounters. Global Health.

[19] Piro FN, Naess O, Claussen B (2007). Area deprivation and its association with health in a cross-sectional study: are the results biased by recent migration?. BMC Health Serv Res.

[20] Tay AK, Rees S, Chen J, Kareth M, Lahe S, Kitau R, et al. Associations of Conflict-Related Trauma and Ongoing Stressors with the Mental Health and Functioning of West Papuan Refugees in Port Moresby, Papua New Guinea (PNG). PLoS One. 2015;10(4):e0125178 10.1371/journal.pone.0125178 PMC4414604 25923209

[21] Lopes Cardozo B, Vergara A, Agani F, Gotway CA (2000). Mental health, social functioning, and attitudes of Kosovar Albanians following the war in Kosovo. JAMA.

[22] Makhashvili N, Chikovani I, McKee M, Bisson J, Patel V, Roberts B. Mental disorders and their association with disability among internally displaced persons and returnees in Georgia. J Trauma Stress. 2014;27(5):509–518 10.1002/jts.21949 PMC4496794 25322880

[23] Hamid AA, Musa SA (2010). Mental health problems among internally displaced persons in Darfur. Int J Psychol.

[24] Mujeeb A: Mental health of internally displaced persons in Jalozai camp, Pakistan. Int J Soc Psychiatry. 2015. [Epub ahead of print] 10.1177/0020764015573083 25770202

[25] Siriwardhana C, Adikari A, Pannala G, Siribaddana S, Abas M, Sumathipala A, Stewart R (2013). Prolonged internal displacement and common mental disorders in Sri Lanka: the COMRAID study. PLoS One.

[26] Mels C, Derulyn I, Broekaert E, Rossel Y (2010). The psychological impact of forced displacement and related risk factors on Eastern Congolese adolescents affected by war. J Child Psychol Psychiatry.

[27] Siriwardhana C, Stewart R (2013). Forced migration and mental health: prolonged internal displacement, return migration and resilience. Int Health.

[28] Siriwardhana C, Ali SS, Robert B, Stewart R (2014). A systematic review of resilience and mental health outcomes of conflict-driven adult forced migrants. Confl Health.

[29] Aggarwal AN, Agarwal R, Gupta D (2014). Abbreviated World Health Organization Quality of Life questionnaire (WHOQOL-Brief) in north Indian patients with bronchial asthma: an evaluation using Rasch analysis. NPJ Prim Care Respir Med.

[30] Kinyanjui DW, Kathuku DM, Mburu JM (2013). Quality of life among patients living with epilepsy attending the neurology clinic at Kenyatta National Hospital, Nairobi, Kenya: a comparative study. Health Qual Life Outcomes.

[31] Diener E, Emmons RA, Larsen RJ, Griffin S (1985). The Satisfaction with Life Scale. J Pers Assess.

[32] Goldberg D, Williams P (1988). A User’s Guide to the General Health Questionnaire.

[33] Abubakar A, Fischer R (2012). The factor structure of the 12-item General Health Questionnaire in a literate Kenyan population. Stress Health.

[34] Ministry of Health, Kenya: Nakuru County. Health at a glance [http://www.healthpolicyproject.com/pubs/291/Nakuru%20County-FINAL.pdf].

[35] National AIDS Control Council. HIV and AIDS profile: Nakuru County [http://www.nacc.or.ke/images/documents/KenyaCountyProfiles.pdf]

[36] De Menil V: Hospital escapees highlight need for community mental health in Kenya. [http://blogs.lse.ac.uk/healthandsocialcare/2013/05/16/hospital-escapees-highlight-need-for-community-mental-health-in-kenya]

[37] BasicNeeds. Kenya. [http://www.basicneeds.org/where-we-work/kenya]

[38] Kiima D, Jenkins R (2010). Mental health policy in Kenya -an integrated approach to scaling up equitable care for poor populations. Int J Ment Health Syst.

[39] Lockhart L. One woman’s dedication to improve mental health treatment and understanding in Kenya. [http://www.mentalhealthy.co.uk/other/mental-health/mental-health-in-kenya.html]

[40] Kenya Human Rights Commission and the National Network for IDPs in Kenya: Gains and Gaps: A status report on IDPs in Kenya 2008–2010. [http://www.khrc.or.ke/resources/publications/doc_download/17-gains-and-gaps-a-status-report-on-idps-in-kenya-2008-2010.html]

[41] Kenya Human Rights Commission. Out in the Cold: The Fate of Internally Displaced Persons in Kenya (2008-2009). Kenya Human Rights Commission, Nairobi: 2009. ISBN: 9966941614.

[42] The Baring Foundation. Baseline Report. The right to information for internally displaced persons in Kenya. [http://www.article19.org/data/files/medialibrary/3559/IDP-Report.pdf]

[43] Internal Displacement Monitoring Centre. Overview. Kenya: too early to turn the page on IDPs, more work is needed. [http://www.internal-displacement.org/sub-saharanafrica/kenya/2014/kenya-too-early-to-turn-the-page-on-idps-more-work-is-needed--]

[44] Field A (2009). Discovering statistics using SPSS.

[45] Sheikh TL, Abdulaziz M, Agunbiade S, Joseph I, Ebiti B, Adekeye O (2015). Correlates of depression among internally displaced persons after post-election violence in Kaduna, North Western Nigeria. J Affect Disord.

[46] Roberts B, Browne J, Ocaka KF, Oyok T, Sondorp E (2008). The reliability and validity of the SF-8 with a conflict-affected population in northern Uganda. Health Qual Life Outcomes.

[47] Ware J, Kosinski M, Dewey J, Gandek B (2001). How to Score and Interpret Single-Item Health Status Measures: A Manual for Users of the SF-8 Health Survey.

[48] Glaesmer H, Grande G, Braehler E, Roth M (2011). The German Version of the Satisfaction with Life Scale (SWLS) Psychometric Properties, Validity, and Population-Based Norms. Eur J Psychol Assess.

[49] Vázquez C, Duque A, Hervás G (2013). Satisfaction with life scale in a representative sample of Spanish adults: validation and normative data. Span J Psychol.

[50] Pavot W, Diener E (2009). Review of the satisfaction with Life Scale. Soc Indi Res.

[51] Frisch MB, Cornell J, Villanueva M, Retzlaff P (1992). Clinical validation of the Quality of Life Inventory: A measure of life satisfaction for use in treatment planning and outcome assessment. Psychol Assess.

[52] Ozbay F, Johnson DC, Dimoulas E, Morgan CA, Charney D, Southwick S (2007). Social support and resilience to stress: from neurobiology to clinical practice. Psychiatry (Edgmont).

[53] Gorst-Unsworth C, Goldenberg E (1998). Psychological sequelae of torture and organised violence suffered by refugees from Iraq. Trauma-related factors compared with social factors in exile. Br J Psychiatry.

[54] Zisook S, Chentsova-Dutton Y, Shuchter SR (1998). PTSD following bereavement. Ann Clin Psychiatry.

[55] Nakajima S, Ito M, Shirai A, Konishi T (2012). Complicated grief in those bereaved by violent death: the effects of post-traumatic stress disorder on complicated grief. Dialogues Clin Neurosci.

[56] Nickerson A, Liddell BJ, Maccallum F, Steel Z, Silove D, Bryant RA (2014). Posttraumatic stress disorder and prolonged grief in refugees exposed to trauma and loss. BMC Psychiatry.

[57] Zisook S, Kendler KS (2007). Is bereavement-related depression different than non-bereavement-related depression?. Psychol Med.

[58] Gilman SE, Breslau J, Trinh NH, Fava M, Murphy JM, Smoller JW (2012). Bereavement and the diagnosis of major depressive episode in the National Epidemiologic Survey on Alcohol and Related Conditions. J Clin Psychiatry.

[59] Caserta MS, Utz RL, Lund DA (2013). Spousal bereavement following cancer death. Illn Crises Loss.

[60] Araya M, Chotai J, Komproe IH, de Jong JT (2007). Effect of trauma on quality of life as mediated by mental distress and moderated by coping and social support among post conflict displaced Ethiopians. Qual Life Res.

[61] Roberts B, Ocaka KF, Browne J, Oyok T, Sondorp E (2008). Factors associated with post-traumatic stress disorder and depression amongst internally displaced persons in northern Uganda. BMC Psychiatry.

